# Functional Expression of Multidrug-Resistance (MDR) Transporters in Developing Human Fetal Brain Endothelial Cells

**DOI:** 10.3390/cells11142259

**Published:** 2022-07-21

**Authors:** Phetcharawan Lye, Enrrico Bloise, Guinever E. Imperio, David Chitayat, Stephen G. Matthews

**Affiliations:** 1 Department of Physiology, University of Toronto, Toronto, ON M5S 1A8, Canada; tlye@lunenfeld.ca (P.L.); ebloise@icb.ufmg.br (E.B.); 2 Lunenfeld-Tanenbaum Research Institute, Sinai Health System, Toronto, ON M5G 1X5, Canada; guinever.imperio@mail.utoronto.ca; 3 Department of Morphology, Federal University of Minas Gerais, Belo Horizonte 31270-910, MG, Brazil; 4 The Prenatal Diagnosis and Medical Genetics Program, Department of Obstetrics and Gynecology, Mount Sinai Hospital, University of Toronto, Toronto, ON M5S 1A8, Canada; dchitayat@mtsinai.on.ca; 5 Division of Clinical and Metabolic Genetics, Department of Pediatrics, The Hospital for SickKids, University Toronto, Toronto, ON M5G 1X8, Canada; 6 Department of Obstetrics and Gynecology, University of Toronto, Toronto, ON M5S 1A8, Canada; 7 Department of Medicine, Temerty Faculty of Medicine, University of Toronto, Toronto, ON M5S 3H2, Canada

**Keywords:** developing human blood–brain barrier (BBB), P-glycoprotein (P-gp/*ABCB1*), breast cancer resistance protein (BCRP/*ABCG2*), fetal brain endothelial cells (BECs), tube formation

## Abstract

There is little information about the functional expression of the multidrug resistance (MDR) transporters P-glycoprotein (P-gp, encoded by *ABCB1*) and breast cancer resistance protein (BCRP/*ABCG2*) in the developing blood–brain barrier (BBB). We isolated and cultured primary human fetal brain endothelial cells (hfBECs) from early and mid-gestation brains and assessed P-gp/*ABCB1* and BCRP/*ABCG2* expression and function, as well as tube formation capability. Immunolocalization of the von Willebrand factor (marker of endothelial cells), zonula occludens-1 and claudin-5 (tight junctions) was detected in early and mid-gestation-derived hfBECs, which also formed capillary-like tube structures, confirming their BEC phenotype. P-gp and BCRP immunostaining was detected in capillary-like tubes and in the cytoplasm and nucleus of hfBECs. P-gp protein levels in the plasma membrane and nuclear protein fractions, as well as P-gp protein/*ABCB1* mRNA and BCRP protein levels decreased (*p* < 0.05) in hfBECs, from early to mid-gestation. No differences in P-gp or BCRP activity in hfBECs were observed between the two age groups. The hfBECs from early and mid-gestation express functionally competent P-gp and BCRP drug transporters and may thus contribute to the BBB protective phenotype in the conceptus from early stages of pregnancy.

## 1. Introduction

The biological events leading to blood–brain barrier (BBB) development are well documented in animal models [1]; however, less is known about how and when human brain endothelial cells (BECs) acquire their important protective phenotype [2]. Newly created blood vessels within the developing brain supply oxygen and nutrients to the surrounding neuronal tissues while removing waste products and promoting immune surveillance [3]. Within the developing brain, newly formed blood vessels may contribute to the BBB providing a mechanism to protect the developing central nervous system (CNS) from harmful substances present in the fetal circulation [4].

The BBB is formed by non-fenestrated mesenchyme-derived endothelial cells, enclosed within brain capillaries ensheathed by astrocyte foot processes and pericytes [5]. Tight junctions form between brain endothelial cells (BECs) to decrease paracellular diffusion and prevent cellular transmigration [6]. This endothelial barrier also contains a variety of transporter systems at the luminal and ab-luminal surface, to control the influx and efflux of a large range of molecules across the barrier. Regulated transport of some compounds across the BBB is important to support normal brain growth and function [7]. However, transporters also function to protect the brain from neurotoxins and xenobiotics (including drugs, chemicals and environmental pollutants)—that may be present in the fetal circulation [8,9].

The multidrug resistance (MDR) transporters, P-glycoprotein (P-gp; encoded by *ABCB1*) and breast cancer resistance protein (BCRP; encoded by *ABCG2*) localized to the BBB prevent the entry of factors into the brain, as well as facilitating the clearance of potentially toxic xenobiotics, from within the brain back into the circulatory system [5,10]. P-gp and BCRP are also highly expressed in the plasma membrane of many barrier-like tissues, including the intestinal [11] and kidney luminal epithelium [12], liver hepatocytes [13,14], the blood–testis barrier [10], as well as the yolk sac [15] and placental barriers [16,17].

P-gp and BCRP are likely developmentally regulated at the BBB [18,19,20]. Previous studies in human cell lines or in animal models have shown them to be modulated by hormones (glucocorticoids) [20], growth factors (transforming growth factor [TGF]-ß) [19,21], cytokines (interleukin [IL]-1β, IL-6, interferon gamma [IFN]-γ or tumor necrosis factor [TNF]-α, [22], as well as viral and bacterial challenge [23]. Of importance, P-gp expression in brain microvessels increases in late gestation in the fetal guinea pig [19,20], and mouse brain [10,24], indicating that brain protection is acquired through pregnancy in these animal models. In humans, P-gp immunostaining in cortical samples identified P-gp in fetal brain in mid and late pregnancy, and that levels increased postnatally [18]. However, there is little information about P-gp and BCRP expression and function in the human fetal BBB, particularly in the early and mid-pregnancy stages.

We hypothesized that the functional expression of P-gp and BCRP in the human fetal brain microvasculature are developmentally regulated. In the present study, we characterized primary human fetal brain endothelial cells (hfBECs) isolated from brain tissue derived during the early and mid- gestation of human pregnancy. We determined their functional expression profile of P-gp and BCRP, as well as their ability to form capillary-like tube structures and express tight junction proteins.

## 2. Materials and Methods

### 2.1. Ethical Approval

Early and mid-gestation fetal brains were collected following elective termination of pregnancies at 12.4 ± 0.7 (*n* = 6; mean ± SD) and 17.9 ± 0.5 (*n* = 6; mean ± SD) weeks of gestation, respectively, by the Research Centre for Women’s and Infants’ Health BioBank program at the Sinai Health System. Written informed consent (protocol #18-0057-E) was obtained in adherence to the policies of the Sinai Health System and the University of Toronto Research Ethics Boards. The REBs do not allow the collection or reporting of any identifying or clinical information from elective pregnancy terminations.

### 2.2. Isolation and Culture of Human Brain Endothelial Cells

The majority of available human fetal brain tissue was used for BEC isolation. However, where possible, a portion of tissue was fixed in 4% paraformaldehyde (PFA; 1570 Electron Microscopy Sciences, Hatfield, PA, USA) for immunofluorescence. Human fetal BECs (hfBECs) were isolated as previously described with adaptations [22]. Briefly, small fragments of fetal brain tissue were placed on ice-cold medium 199 (12340030, ThermoFisher Scientific, Waltham, MA, USA) and supplemented with penicillin (100 IU/mL) and streptomycin (100 IU/mL; 15140-122, Life Technologies, Carlsbad, CA, USA). All remaining steps took place under sterile conditions. Brain fragments were minced and the resulting tissue was centrifuged (1500 rpm, 5 min, 4 °C). The pellet was resuspended in dextran (d8821, Sigma-Aldrich, St. Louis, MO, USA) solution (17.5% *w*/*v* dextran in Hank’s Balanced salt solution (HBSS++) and centrifuged (2500 rpm, 25 min, 4 °C). The pellet (containing the microvessel fraction) was resuspended in HBSS++ containing collagenase (c5138-1g,1 mg/mL; Sigma-Aldrich) and digested (20 min, 37 °C). The mixture was then centrifuged (2500 rpm, 10 min, 4 °C), and the cell pellet resuspended in warm 199 media supplemented with 20% fetal bovine serum (FBS; 080450, Wisent, St Bruno, QC, Canada). Cells were plated on type I rat tail collagen (50 μg/mL; 5056, Advanced BioMatrix, San Diego, CA, USA) coated tissue culture flasks (353136, ThermoFisher Scientific). The hfBECs were grown in a 37 °C/5% CO_2_-incubator in EndoGROTM-MV Complete Culture Media Kit^®^, (SCME004, Millipore, Blvd, ON, Canada) and supplemented with recombinant human epidermal growth factor (5 ng/mL) L-Glutamine (10 mM), hydrocortisone hemisuccinate (1.0 µg/mL), heparin sulfate (0.75 U/mL), ascorbic acid (50 µg/mL), 20% FBS, penicillin (100 IU/mL), streptomycin (100 IU/mL) (15140-122, Life Technologies) and 1% normocin antibiotic (ant-nr-2, Invivogen, San Diego, CA, USA) at 20% O_2_ (5% CO_2_, 37 °C). The hfBECs were collected and stored in liquid nitrogen. Due to the limited number of cells available, hfBEC culture experiments were undertaken at passage 4 for all experiments.

Adult human cerebral microvascular endothelial cell line (hCMEC/D3) cells (30 passages; CLU512, Cedarlane Labs, Burlington, ON, Canada) were cultured as previously described [25]. Briefly, hCMEC/D3 cells were cultured at 20% O_2_ (5% CO_2_, 37 °C) with EndoGRO™-MV Complete Culture Media Kit^®^ (SCME004, Millipore), human basic fibroblast growth factor (1 ng/mL; F0291, Sigma) and 1% penicillin-streptomycin (10,000 units-10,000 μg/mL, 15140-122, Life Technologies) for 24 h.

Human Brain Vascular Pericytes cell line (HBVP) cells (4 passages; 1200, Sciencell Research Laboratories, Carlsbad, Canada) were cultured as previously described [26]. Briefly, HBVP cells were cultured at 20% O_2_ (5% CO_2_, 37 °C) with pericyte medium (PM; 1201, ScienCell Research Laboratories) for 24 h.

Human fetal brain astrocytes (hfBAST) were isolated as previously described with adaptations [27]. Briefly, brain tissue was cut into small pieces and digested in cocktail enzyme collagenase (2 mg/mL, C6885-1g, Sigma), soy bean trypsin inhibitor (0.15 mg/mL, T9003-250MG, Sigma), DNAse I (0.15 mg/mL, Roche, Mississauga, ON, Canada), albumin (BSA; 1 mg/mL, ALB001.500, BioShop, Burlington, ON, Canada) and 1% FBS in HBSS−/−) [28] in a 37 °C bead bath for 20 min. After incubation, cocktail enzyme was removed by centrifugation (1000× *g*, 10 min), the pellet was resuspended in Neurobasal A medium (10888022; Gibco Life Technologies) and loaded onto a column containing glass beads (0.5 mm; Z250465, Sigma) and filter (SX0002500; EMD Millipore, Billerica, MA, USA). The filtrate was plated on type I rat tail collagen (50 μg/mL; Advanced BioMatrix) in 75 mm^2^ tissue culture flasks (BD Biosciences) and cultured in 20% O_2_ (5% CO_2_, 37 °C) with DEMEM/F12 (1:1) (11039-021, Gibco Life Technologies), 10% FBS, penicillin (100 IU/mL), streptomycin (100 IU/mL) (Life Technologies), 1% normocin antibiotic (Invivogen). The hfBAST culture experiments were undertaken.

### 2.3. Immunofluorescence

Immunofluorescence experiments were performed as described previously [29]. In brief, brain tissue was sectioned (5 µm), deparaffinized, rehydrated and subjected to antigen retrieval with sodium citrate. Autofluorescence was reduced using 0.1% Sudan Black (12806DE, Sigma) in 70% ethanol (1 min) and non-specific binding was blocked using 0.1% BSA (BioShop), 0.3% Triton X-100 (X100-1L, lab grade, Sigma) and 1% donkey serum (017-000-121, Jackson, West Baltimore Pike West Grove, Panama) in PBS (1 h). Brain tissue sections were incubated with primary antibodies, P-gp (SC-55510, 1;100 Santa Cruz Biotechnology, Dallas, TX, USA), BCRP (MAB146, 1:100 in blocking solution, Millipore) and von Willebrand factor (vWF) (ab11713,1:500, Abcam, Toronto, ON, Canada) overnight at 4 °C. The hfBECs were rinsed with cold PBS, fixed with 4% PFA (Electron Microscopy Sciences) for 15 min and then permeabilized with Triton X-100 (lab grade, Sigma) (0.2%, 5 min, room temperature). Autofluorescence was reduced using 0.1% Sudan Black in 70% ethanol (1 min) and non-specific binding was blocked using 1% BSA for 1 h. The hfBECs slides were incubated with primary P-gp, BCRP and vWF antibodies described above, as well as glial fibrillary acidic protein (GFAP), (36705,1:300, Cell Signalling Technology, Danvers, MA, USA) and Lamin B1 (sc-6217,1:200, Santa Cruz Biotechnology, Dallas, TX, USA) overnight at 4 °C. For ZO-1 (339100, 1:200, Invitrogen, ON, Canada) immunofluorescence cells were permeabilized with 0.5% Triton X-100 (lab grade, Sigma) for 15 min and blocked using 2% BSA (BioShop) for 1 h. For Claudin5 (4C3C2, 1:500, Invitrogen) immunofluorescence, cells were fixed with methanol for 10 min and permeabilized with 0.1% Triton X-100 (lab grade, Sigma) for 10 min and blocked using 3% BSA (BioShop) for 10 min and incubated with primary overnight at 4 °C. For immunofluorescence, following the tube formation (see below), cells were fixed 4% PFA (Electron Microscopy Sciences) for 20 min and permeabilized with 0.5% Triton X-100 (lab grade, Sigma) for 15 min and blocked using 2% BSA (BioShop) for 1 h and incubated with primary antibody overnight at 4 °C. Subsequently, cells for (P-gp + vWF), (BCRP + vWF), (BCRP + vWF), (BCRP + Lamin B1) and GFAP immunofluorescence were washed three times and incubated with fluorescent secondary antibodies, the anti-mouse Alexa 488 (A21202, 1:1000), or the anti-goat Alexa 594 (A11058, 1:1000) or the anti-sheep Alexa 555 (A21436, 1:1:000) secondary antibodies (Thermo Fisher Scientific) and counterstained with DAPI (1 μg/mL, 1 h). Fluorescent microscopy was performed using a spinning disc confocal microscope at various magnifications (Leica DMI6000 B, Concord, ON, Canada).

### 2.4. Cellular Fractionation

The hfBECs from early and mid-gestation were grown to confluency in 10 cm^2^ plates and used to determine the subcellular localization of P-gp and BCRP. Isolation of plasma membrane, cytosol, organelles, and nuclei fractions was performed using the Minute™ Plasma Membrane, Protein Isolation and Cell Fractionation Kit (SM-005, Invent Biotech, Plymouth, MN, USA), according to the manufacturer’s protocol (Invent Biotech).

### 2.5. Immunoblotting

Western blot analysis was performed as previously described [16,30]. Briefly, protein isolated from cultured cells was extracted by sonication using lysis buffer (1 mol/L Tris-HCL pH 6.8, 2% SDS, 10% glycerol), which included a protease and phosphatase inhibitor cocktail (78420, Thermo Scientific). The protein concentration was determined with the Pierce BCA Protein Assay kit (Thermo Scientific). Proteins were separated by electrophoresis (20 μg or 30 μg 100 V, 1 h) using SDS polyacrylamide gels (8% or 12%). Proteins were then transferred (10 min) to polyvinylidene fluoride (PVDF) membrane using Trans-Blot Turbo (Bio-Rad, Hercules, CA, USA). Membranes were blocked with skim milk (5%; 1 h, room temperature). The primary antibodies used were P-gp (ab170903, dilution 1:1000; Abcam, Toronto, ON, Canada), BCRP (ab108312, dilution 1:1000; Abcam), Early Endosome Antigen 1 (EEA1) (C45B10, 1:3000, Cell signaling, Danvers, Massachusetts, USA), Prolyl 4-hydroxylase 3 (P4H3; ab76020, 1: 500, Abcam), Histone3 (H3; ab1791, 1:1000, Abcam), Sodium/potassium ATPase (Na+/K+-ATPase; ab76020,1;30,000, Abcam,) and ERK2 (sc-1647, dilution 1:2000; Santa Cruz Biotechnology). Blots were incubated with primary antibodies overnight (4 °C). The PVDF membranes were subsequently incubated with an HRP-linked anti-rabbit and an anti-rabbit secondary antibody (1:10,000, 1 h; GE Healthcare Bio-Science, Baie d’Urfe, QC, Canada). Protein–antibody complexes were detected by incubating the PVDF membranes with Laminate Crescendo Western HRP Substrate (5 min; Millipore) and chemiluminescence was detected under UV using the ChemiDoc™ MP Imaging system (Bio-Rad). The protein band intensity was quantified using Image Lab™ software.

### 2.6. Quantitative Real Time PCR (qPCR)

Total RNA was isolated from hfBECs using the RNeasy Plus Universal Mini Kit (73404, Qiagen, Toronto, ON, Canada), as previously described [16,31]. RNA concentration and purity were assessed using a NanoDrop1000 Spectrophotometer (Thermo Scientific). RNA was reverse transcribed into cDNA using the iScript Reverse Transcription Supermix (Bio-Rad). *ABCB1* and *ABCG2* mRNA levels were measured by qPCR using SYBR Green reagent (Sigma-Aldrich) and the CFX 380 Real-Time system C1000 TM Thermal Cycler (Bio-Rad), with the following cycling conditions: initial denaturation at 95 °C (2 min) followed by 39 cycles of denaturation at 95 °C (5 s) and combined annealing and extension at 60 °C (20 s). Gene expression was normalized to the geometric mean of DNA topoisomerase 1 (*TOP1*) and the zeta polypeptide (*YWHAZ*), which exhibited stable expression. The relative expression of target genes was calculated by the 2-ΔΔCT method [32]. The primer sequences for all the assessed genes are provided in Table 1.

### 2.7. P-gp and BCRP Activity Assay

P-gp function was assessed, as described previously [20], with adaptations. Briefly, hfBECs or adult human brain endothelial cell line (hCMEC/D3) were seeded as described above. Cells washed twice with warm Tyrode salts’ solution (T2145, Sigma) supplemented with sodium bicarbonate (1 g/L; S6014, Sigma). Cells were incubated with P-gp substrate calcein-acetoxymethyl ester (Ca-AM, 177831, 10^−6^ M, Sigma) with or without a specific P-gp inhibitor, verapamil (V4629, VPL, 10^−4^ M, Sigma; 37 °C, 5% CO_2_, 1 h). Ca-AM is intracellularly cleaved by endogenous esterases, producing fluorescent calcein that cannot be transported by P-gp and, therefore, serves as an indirect measure of P-gp function. After incubation with Ca-AM, the plates were placed on ice and the cells were washed twice with ice-cold Tyrode salts’ solution (Sigma), followed by cell lysis with 1% Triton X-100 (X100, Sigma) lysis buffer. Cellular content of Ca-AM was measured with a fluorescent microplate reader at excitation and emission wavelengths of 485 nm and 510 nm. Ca-AM accumulation was normalized to protein. P-gp function was also verified using a rhodamine 123 accumulation assay (Rho123, R302, Thermo Scientific). Cells were washed before incubation with Rho123 (10^−5^ M) in the presence or absence of VPL (10^−4^ M) for 45 min. After lysis, Rho123 accumulation was measured at excitation and emission wavelengths of 485 nm and 528 nm [19,20]. Rho123 accumulation was normalized to protein.

BCRP function was assessed, as previously described [34,35], with adaptations. Briefly, hfBECs were seeded as described above. The BCRP substrate Chlorin e6 (Ce6; 2 uM; SC-263067, Santa Cruz Biotechnology) was pre-incubated with Tyrode salts’ solution (Sigma) in a 37 °C bead bath for 30 min in the presence or absence of the specific BCRP inhibitor, Ko143 (K2114, 10 μM, Sigma). Ce6 solution (with or without Ko143) was then loaded into hfBECs (37 °C, 5% CO_2_, 1 h). After incubation with Ce6, the plates were placed on ice and the cells were washed twice with ice-cold Tyrode salts’ solution (Sigma), followed by cell lysis with 1% Triton X-100 (X100, Sigma) lysis buffer. Cellular content of Ce6 was measured with a fluorescent microplate reader at excitation and emission wavelengths of 407 nm and 667 nm. Ce6 accumulation was normalized to protein.

### 2.8. Matrigel Tube Formation Assay

Tube formation was performed, as previously described, with adaptations [36]. Briefly, Matrigel basement membrane matrix (354262, Corning, Bedford, MA, USA) was thawed and placed on ice until use. A 30 μL aliquot was dispensed onto each well of a chilled 96-well culture plate using pre-cooled pipet tips. The plate was incubated (37 °C, 10–15 min) to allow the Matrigel to solidify. The hfBECs (30,000 cells/100 μL DMEM containing 2% FCS) were added to each well. The plate was incubated at 37 °C and 5% CO_2_, and tube formation was observed at 10× magnification at 24 h using the QiMAGING micropublisher 5.0 RTV (Leica, Wetzlar, Germany).

### 2.9. Cell Proliferation and Viability

Proliferation and viability of early and mid-pregnancy hfBECs were determined by XTT dye-reduction assay and trypan blue cell exclusion, respectively, as previously described [37,38]. For the cell proliferation assay, 5000 cells/well were plated into 96-well plates and maintained in culture for 3 days. On the day of assay at 24, 48 and 72 h, 50 μL of XTT (5 mg/mL; X6493, Invitrogen, Burlington, ON, Canada) and 0.4 μL of phenazine methosulfate (1 mg/mL; PSM; Sigma) were added to each well and the cells were incubated (37 °C for 3 h). Absorbance was measured at 450 nm. For cell viability assessment, cells (15,000/well) were seeded into 6-well plates. Trypan blue was added at 24, 48 and 72 h, and the ratio of cells containing dye vs those that did not was calculated.

### 2.10. Statistical Analyses

Data analyses were performed with Prism version 8 (GraphPad Software Inc., San Diego, CA, USA). Outliers were identified using the ROUT method and normality was assessed using D’Agostino–Pearson test. Following normality testing, differences between the Ca-AM, Rho123 and Ce6 accumulation, P-gp/*ABCB1* and BCRP/*ABCG2* protein, mRNA expression and subcellular fractions in hfBECs were determined using a paired *t*-test or unpaired t-test. For hCMEC/D3, analysis was by un-paired *t*-test. For the proliferative assay and Trypan Blue cell viability, two-way repeated measures ANOVA followed by Sidak’s multiple comparisons test was undertaken. Differences were considered statistically significant at *p* ≤ 0.05.

## 3. Results

### 3.1. Human Fetal Brain Endothelial Cells (hfBECs) Derived from Early and Mid-Gestation Express the Endothelial Cell Maker von Willebrand Factor (vWF)

**Figure 1:**
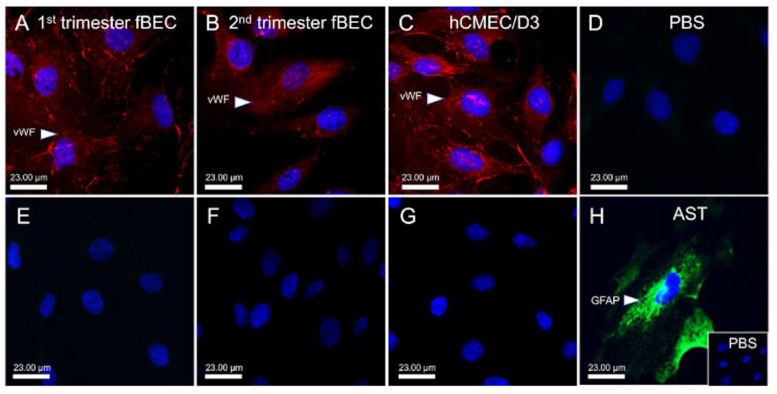
**Expression of von Willebrand Factor (vWF) in early and mid-gestation cultured primary human fetal brain endothelial cells (hfBECs).** (**A**–**C**) Representative immunofluorescence of the endothelial cell marker von Willebrand factor (vWF; red) identified the hfBECs in the early (**A**) and mid- (**B**) gestation and in the adult endothelial hCMEC/D3 cell line (**C**). (**A**–**H**) DAPI (blue; a nuclear marker). (**E**–**G**) Glial fibrillary acidic protein (GFAP; green—a glial cell marker) staining was absent in all hfBEC cultures. (**H**) GFAP positive staining was confirmed in primary human fetal brain astrocyte culture (**D**) PBS (negative control). *n* = 4/gestational age (representative images presented). Scale bar represents 23 μm.

We examined the vWF (marker of endothelial cells) immunolocalization in primary hfBECs and in fetal brain microvessels derived from human fetal brain tissue at mid-pregnancy, as well as in adult hCMEC/D3 cells as positive controls. The vWF displayed prominent staining within microvessels in the fetal brain (Supplementary Figure S1A–C) and the luminal plasma membrane and cytoplasm of early and mid-gestation hfBECs, as well as in the adult endothelial cell line hCMEC/D3 (Figure 1A–C). Early and mid-gestation hfBECs and adult hCMEC/D3 cells did not stain positive for the astrocyte marker, GFAP (Figure 1E–G). In contrast, primary hfBAST cells stained positive for GFAP (Figure 1H).

### 3.2. Expression of Tight Junction Proteins by hfBECs in Early and Mid-Gestation

The expression of zonula occludens-1 (ZO-1) and claudin-5 (Cldn-5) proteins in early and mid-gestation hfBECs was assessed by immunofluorescence. The expression of both ZO-1 and Cldn-5 was evident in the cytoplasm and plasma membrane of early (Supplementary Figure S2A,E) and mid-gestation hfBECs (Supplementary Figure S2B,F). Similar staining in adult hCMEC/D3 cells (Supplementary Figure S2C,G) further supports the endothelial nature of the derived fetal primary cells.

### 3.3. Tube Formation by Early and Mid-Gestation Derived hfBECs, and Expression of Endothelial Markers and Drug Transporters

**Figure 2:**
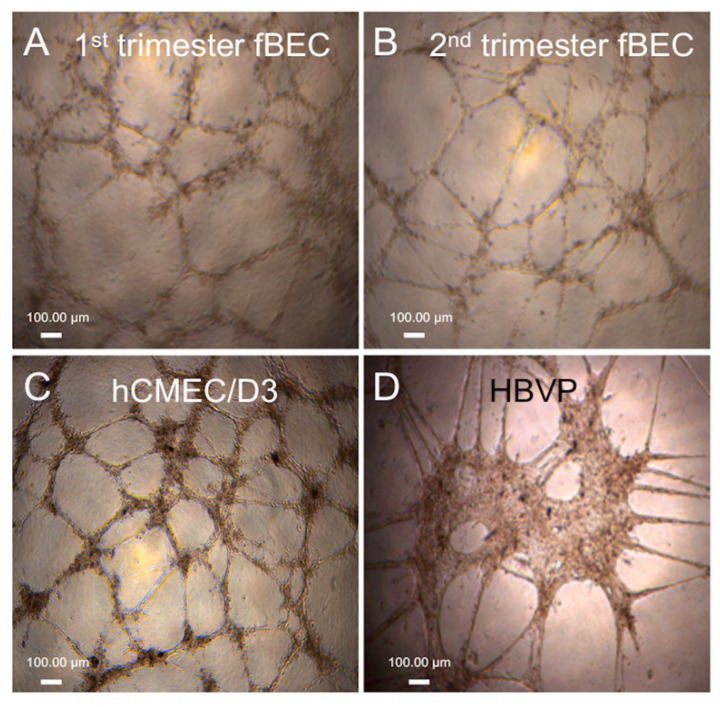
**Tube formation in early and mid-gestation primary human fetal brain endothelial cells (hfBECs)**. (**A**) early pregnancy hfBECs, (**B**) mid-pregnancy hfBECs, (**C**) adult endothelial cell line (hCMEC/D3) as positive control, and (**D**) human brain vessel pericyte cell line (HBVP). Tube formation was assessed after 24 h. *n* = 6/gestational age (representative images presented).

We tested the ability of the isolated early and mid-gestation hfBECs to form capillary-like tube structures in vitro over a 24 h period, with adult hCMEC/D3 cells serving as a positive control. Visual assessment identified that both hfBECs and hCMEC/D3 formed similar tube structures in vitro over the 24 h period (Figure 2A–C). Structural formations were quite different in shape and more slowly forming than those formed by the human brain vessel pericyte cell line (HBVP; Figure 2D). Thus, confirming a BEC phenotype of the isolated hFBECs.

**Figure 3:**
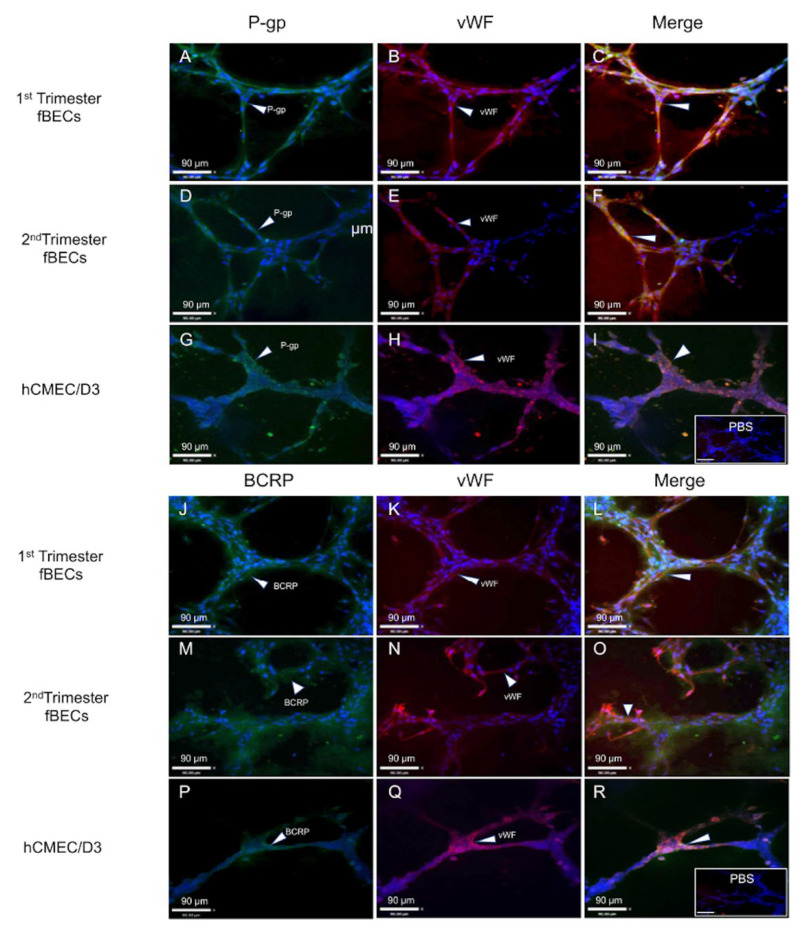
**P-glycoprotein (P-gp), breast cancer resistance protein (BCRP) and von Willebrand factor (vWF) are expressed by capillary-like tube structures formed by early and mid-gestation primary human fetal brain endothelial cells (hfBECs).** Representative immunofluorescence images of P-gp (**A**,**D**,**G**) BCRP (**J**,**M**,**P**) and vWF (**B**,**E**,**H**,**K**,**N**,**Q**) in early gestation (**A**–**C**, **J**–**L**), and mid-gestation (**D**–**F**, **M**–**O**) hfBECs and in the adult endothelial cell line hCMEC/D3 (as positive control) (**G**–**I**, **P**–**R**). Arrows indicate P-gp, BCRP and vWF staining within capillary-like tube structures. Expression of vWF (red) confirmed the endothelial cell phenotype. P-gp and BCRP (green) and vWF (red) staining co-localize (yellow) inside the capillary-like tube structures. Insets (bottom right) in (**I**,**R**) represent PBS as the negative control. Sections were counter-stained with DAPI (blue; a nuclear marker). *n* = 4/gestational age (representative images presented). Scale bars = 90 um.

We next examined the expression and immunolocalization of the drug transporters P-gp and BCRP and the endothelial cell marker vWF in the tube structures formed by early and mid-gestation hfBECs and hCMEC/D3 (as control). As expected, these endothelial cells exhibited prominent staining for P-gp (Figure 3A,D) and BCRP (Figure 3J,M) within the tube structures formed by both early and mid-gestation hfBECs, as was also the case for hCMEC/D3 cells (P-gp; Figure 3G and BCRP; Figure 3P). Furthermore, these cells also exhibited the expression of the endothelial cell marker vWF within the tubes formed by these endothelial cells (Figure 3B,E,H,K,N,Q). Merged images confirmed co-expression of the drug transporters with the endothelial cell marker within the tube structures formed by early and mid-gestation hfBECs (Figure 3C,F,L,O) and by the hCMEC/D3 cells (Figure 3I,R).

### 3.4. Expression and Localization of P-gp and BCRP in the Human Fetal Brain Tissue and in Primary Human Fetal Brain Endothelial Cells (hfBECs)

**Figure 4:**
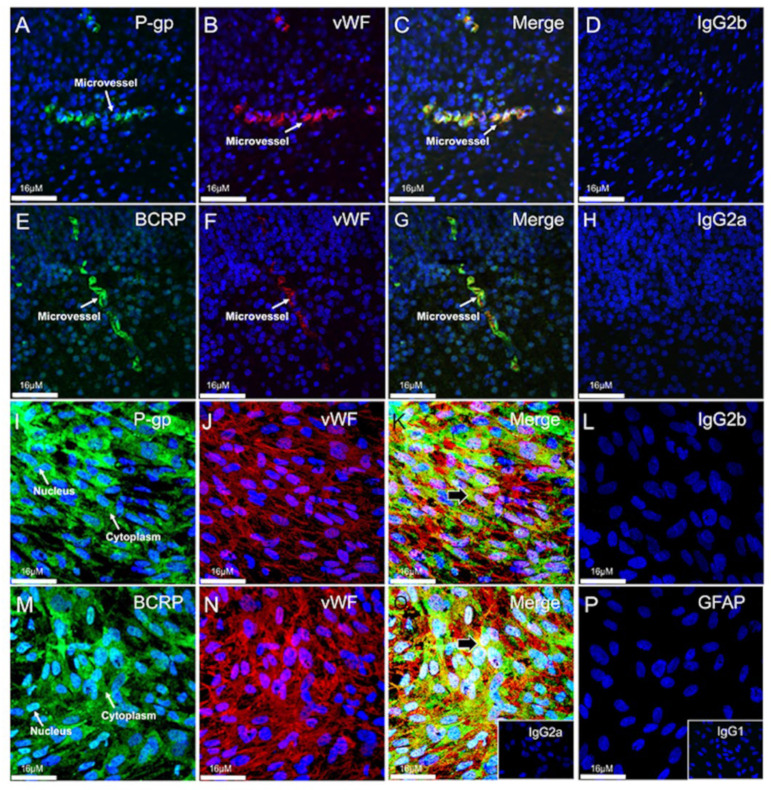
**P-glycoprotein (P-gp) and breast cancer resistance protein (BCRP) are enriched in mid-gestation human brain microvessels and in mid-gestation primary human fetal brain endothelial cells (hfBECs).** Representative immunofluorescence (**A**–**H**) images of P-gp and BCRP staining (green) in mid-gestation human fetal brain sections (*n* = 4). (**A**) P-gp and (**E**) BCRP were localized predominantly in the cytoplasm of fetal brain microvessels. (**B**,**F**) Expression of the endothelial cell marker von Willebrand factor (vWF; red) identified the human fetal brain microvessels. (**C**,**G**) Co-localization of P-gp, BCRP and vWF staining confirmed P-gp and BCRP localization within the endothelium of human fetal brain microvessels. (**I**–**P**) Representative immunofluorescence images of cultured mid-gestation hfBECs. (**I**) P-gp (green), (**M**) BCRP (green) staining was localized predominantly in the cytoplasm and nuclei of hfBECs. (**J**,**N**) vWF (red) identified human hfBECs. (**K**,**O**) Co-localization (indicated by black arrows) of P-gp, BCRP and vWF staining confirmed P-gp and BCRP localization within hfBECs. (**L**) IgG2b isotype control. (**P**) Lack of glial fibrillary acidic protein (GFAP) staining confirmed the absence of astrocytes in hfBEC cultures. (**A**–**P**) DAPI (blue; a nuclear marker) or co-staining (yellow). (**H**) IgG2b isotype control. Inserts (bottom right) in (**O**); IgG2a, and (**P**); IgG1 isotype control. (*n* = 4). Scale bar = 16 μm (**A**–**P**).

We assessed P-gp and BCRP immunolocalization within tissue sections obtained from human fetal brains at 18–20 weeks of gestation. Immunofluorescence revealed that the expression of P-gp and BCRP was localized to microvessels within the fetal brain (Figure 4A,E). As expected, P-gp and BCRP displayed prominent staining within the luminal plasma membrane of endothelial cells within microvessels. The fetal brain microvessels stained positive for vWF (an endothelial cell marker; Figure 4B,F), and there was co-localization of P-gp and BCRP with vWF (Figure 4C,G).

Next, we assessed P-gp, BCRP and vWF immunofluorescence in (hfBECs). P-gp and BCRP were localized within the cytoplasm and the nucleus of mid-gestation hfBECs (Figure 4I,M), which also expressed vWF (Figure 4J,N), confirming their endothelial phenotype. Furthermore, hfBECs did not stain for GFAP (marker of glial cells) providing further evidence of their endothelial phenotype (Figure 4P). Merged staining indicated that most but not all vWF positive cells were also positive for P-gp and BCRP and vWF (Figure 4K,O).

### 3.5. P-gp and BCRP Are Enriched in Cytoplasm and Nucleus of Primary Human Fetal Brain Endothelial Cells (hfBECs)

**Figure 5:**
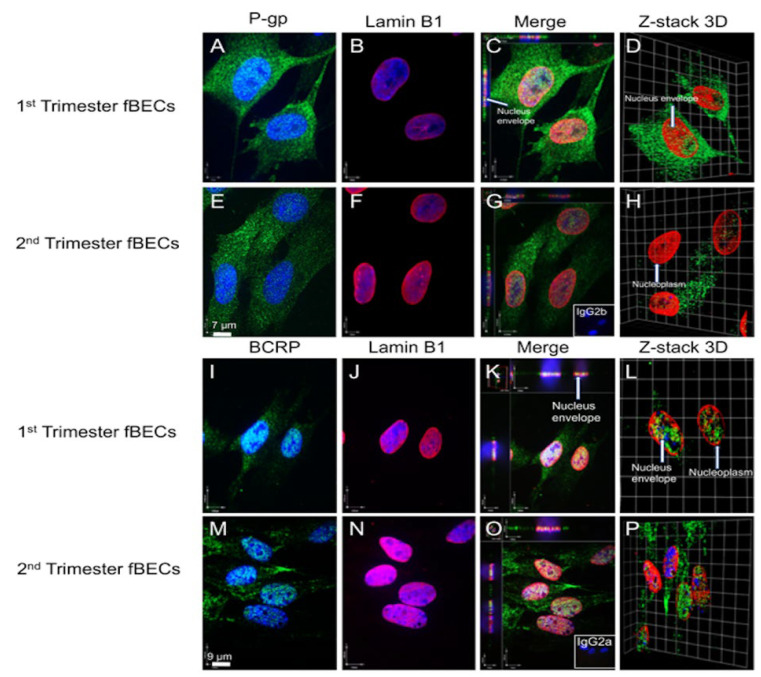
**Expression of P-glycoprotein (P-gp) and breast cancer resistance protein (BCRP) in early and mid-gestation primary human fetal brain endothelial cells (hfBECs)**. Representative immunofluorescence images of P-gp (**A**,**E**) and BCRP (**I**,**M**) subcellular localization in early (**A**–**D**, **I**–**L**) and mid-gestation (**E**–**H**, **M**–**P**) hfBECs. Arrows show P-gp and BCRP staining within nuclear compartments. Lamin B1 staining (red) is used as a nuclear membrane marker. P-gp and BCRP (green) and lamin B1 staining co-localize inside the nucleus. Insets (bottom right) in (**G**) represent the isotype control IgG2b and (**O**) the isotype control IgG2a. (**D**,**H**) Z-stack 3D images of P-gp and Lamin B1 localization and (**L**,**P**) Z-stack 3D images of BCRP and Lamin B1 localization to the nucleus in hfBECs. Sections were counter-stained with DAPI (blue; a nuclear marker). *n* = 3/gestational age (representative images presented). Scale bars = 7 um (P-gp); = 9 μm (BCRP).

We next assessed the subcellular localization of P-gp (Figure 5A,E) and BCRP (Figure 5I,M) in hfBECs derived from both early and mid-gestation fetal brains. Visual examination identified the presence of P-gp and BCRP signals in the cytoplasm and nucleus of these cells. Lamin B1 was used as a marker of the nuclear membrane (Figure 5B,F,J,N). Incubation of sections with both anti-P-gp and BCRP and anti-Lamin B1 confirmed the co-localization of P-gp and BCRP to the cytoplasm, the nuclear membrane and within the nucleus of hfBECs from both the early and mid-gestation fetal brain (Figure 5C,G,K,Q). To further confirm the nuclear localization of P-gp and BCRP, we used confocal microscopy to construct a 3D z-stack through the hfBECs. This clearly showed the presence of P-gp and BCRP within the nucleus of hfBECs from both the early and mid-gestation (Figure 5D,H,L,P).

**Figure 6:**
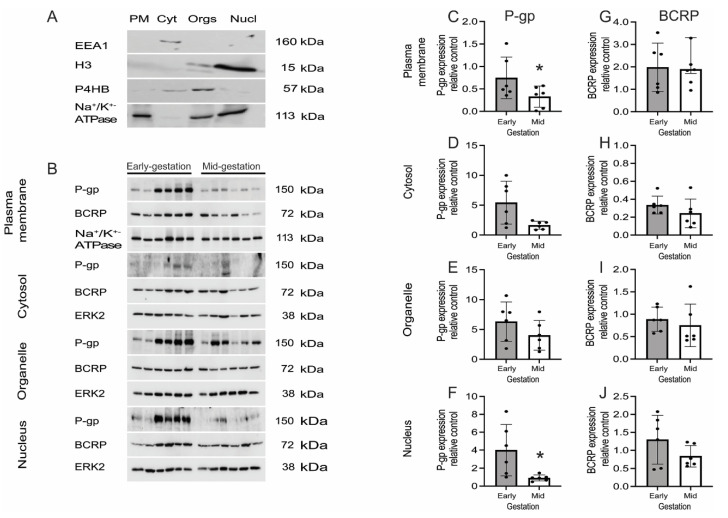
**P-gp and BCRP distribution within subcellular fractions of early and mid-gestation hfBECs.** (**A**) Representative western blot images showing specific markers in subcellular fractions of second trimester hfBECs: plasma membrane (PM, lane 1), cytosol (Cyt, lane 2), organelles (Orgs, lane 3), and nuclear (Nucl, lane 4) fractions. Sodium/potassium ATPase (Na^+^/K^+^-ATPase; plasma membrane marker), early endosome antigen 1 (EEA1; cytosol marker), prolyl 4-hydroxylase 3 (P4H3; endoplasmic reticulum (ER) marker) and histone3 (H3; nuclear marker), *n* = 3. (**B**) Representative western blot images and (**C**–**F**) densitometric analysis of plasma membrane, cytosolic, organelle (ER) and nuclear P-gp expression and (**G**–**J**) densitometric analysis of plasma membrane, cytosolic, organelle (ER) and nuclear BCRP expression, normalized to Na^+^/K^+^-ATPase (plasma membrane loading control) or ERK2 (loading control for cytosol, organelle and nucleus), in early and mid-gestation hfBECs. Data are expressed as mean ± SD. *n* = 6/group. Statistical differences were tested using an unpaired *t*-test. * *p* ≤ 0.05.

In order to confirm and expand our previous immunofluorescence findings showing the presence of P-gp and BCRP in subcellular compartments, we examined the subcellular expression of P-gp and BCRP. The expression of P-gp and BCRP protein within subcellular fractions of early and mid-gestation hfBECs was quantified by Western blotting. We first characterized the subcellular fractions using specific markers that detect the plasma membrane, cytosol, organelles and nuclear fractions of mid-gestation hfBECs (Figure 6A). There was a significant decrease in P-gp in the plasma membrane and nuclear protein fractions (*p* ≤ 0.05) between early and mid-gestation hfBECs (Figure 6C,F) while there was a trend for decreased P-gp levels in the cytosol (*p* = 0.07) and no significant difference in P-gp expression in the organelles (Figure 6D,E). There were no changes in the expression of BCRP between early and mid-gestation in any of the subcellular fractions assessed (Figure 6G–J).

### 3.6. Expression but Not Function of P-gp/ABCB1 and BCRP/ABCG2 Is Developmentally Regulated in Human Fetal Brain Endothelial Cells (hfBECs)

**Figure 7:**
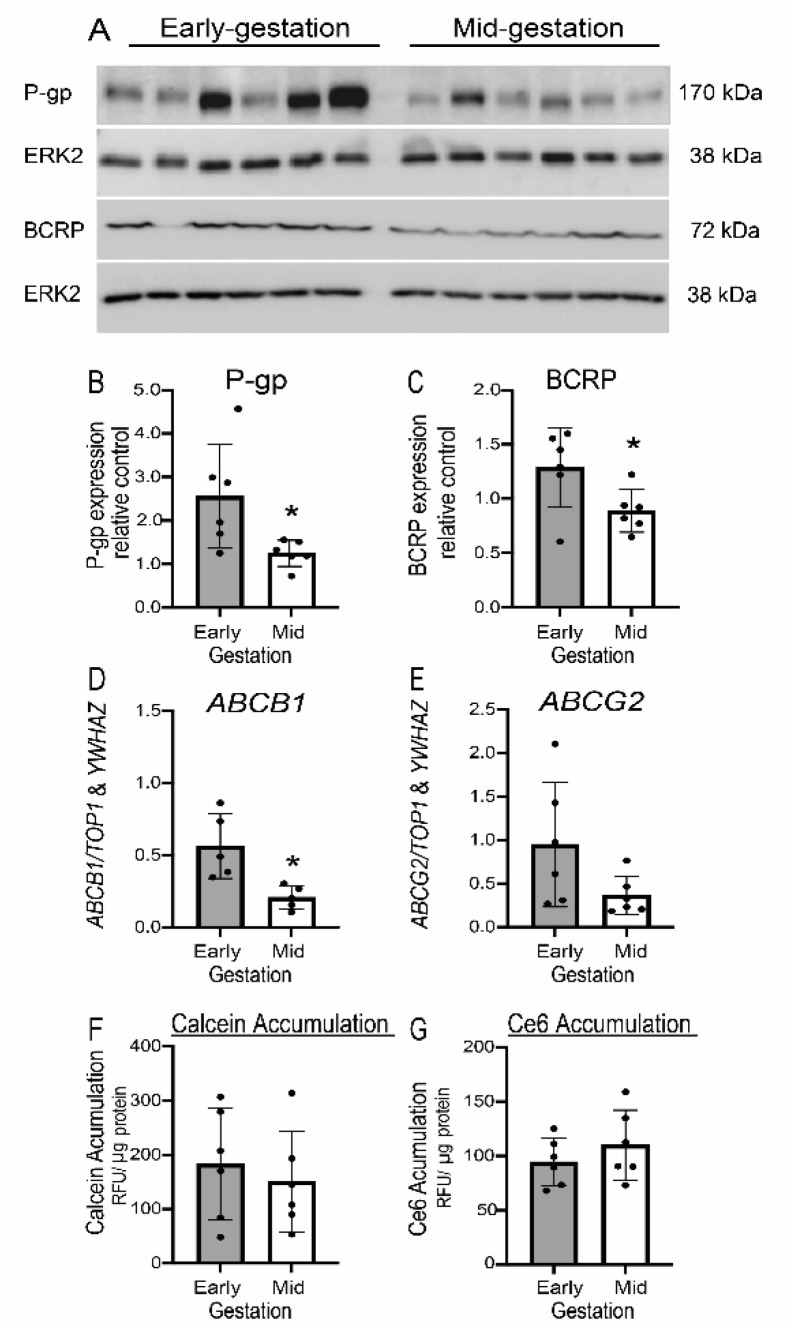
**Developmental expression and function of P-glycoprotein (P-gp/*ABCB1*) and breast cancer resistance protein (BCRP/*ABCG2*) in early and mid-gestation primary human fetal brain endothelial cells (hfBECs).** (**A**) Representative images of western blots (**B**) densitometric analysis of total P-gp and (**C**) BCRP protein comparing early and mid-gestation hfBECs. ERK2 is used as loading control. (**D**) Relative *ABCB1* (encoding P-gp) and (**E**) *ABCG2* (encoding BCRP) mRNA expression in early and mid-gestation hfBECs. Data are normalized by the geometric mean of *TOP1* and *YWHAZ* (reference genes), (**F**) calcein-AM (P-gp substrate) and (**G**) Ce6 (BCRP substrate) accumulation in early and mid-gestation hfBECs. Data are expressed as mean ± SD. *n* = 6/gp. Statistical differences were conducted using an unpaired *t*-test. * *p* ≤ 0.05.

The total protein and mRNA expression of P-gp/ABCB1 and BCRP/ABCG2 in early and mid-gestation hfBECs was measured by western blot and qPCR, respectively. We observed a significant decrease in P-gp protein/*ABCB1* mRNA and BCRP protein (*p* < 0.05) from early to mid-gestation (Figure 7B–D), whereas there was a decreasing trend in *ABCG2* mRNA expression (*p* = 0.08) (Figure 7E). We assessed calcein-AM (P-gp-specific substrate) and Ce6 (BCRP-specific substrate) accumulation in early and mid-gestation hfBECs, as well as in hCMEC/D3 cells (adult BEC line). There was no difference in calcein-AM and Ce6 accumulation between early and mid-gestation hfBECs (Figure 7F,G). VPL (a P-gp inhibitor), increased calcein-AM accumulation in early (*p* ≤ 0.05) and mid-gestation (*p* ≤ 0.01) (Supplementary Figure S3A,B). Ko143 (a BCRP inhibitor) increased Ce6 accumulation in early (*p* ≤ 0.05) and in early and mid-gestation (*p* ≤ 0.001) (Supplementary Figure S3C,D) hfBECs, validating the functional assays. An alternative P-gp substrate, Rho123, was used to confirm the developmental calcein-AM data (Supplementary Figure S4). There was no difference in Rho123 accumulation between early and mid-gestation hfBECs (Supplementary Figure S4A). VPL treatment resulted in the increased cellular accumulation of Rho123 in early (*p* ≤ 0.001) and early and mid-gestation (*p* ≤ 0.01) validating the functional assay (Supplementary Figure S4B,C).

### 3.7. Human Fetal Brain Endothelial Cells (hfBECs) Proliferation and Viability Does Not Change from Early to Mid-Pregnancy

We assessed cell proliferation and viability in order to determine whether there are gestational-age dependent patterns in cell proliferation and viability. We did not detect any gestational-age (group) effect in the proliferation and viability of hfBECs; however, we detected an effect of time in culture in the XTT proliferation assay from 24–72 h (*p* < 0.01), whereas no effect of time was detected in the viability assay (Supplementary Figure S5A,B).

## 4. Discussion

We have characterized fetal endothelial cells isolated from early and mid-gestation human fetal brains and determined the developmental profile of P-gp and BCRP expression and function. von Willebrand Factor (marker of endothelial cells), ZO-1 and Cldn-5 (tight junctions) were highly expressed in hfBECs derived from early and mid-gestation, confirming a BEC phenotype. The hfBECs also formed capillary-like tube structures, very similar to those formed by an adult-derived BEC cell line. P-gp and BCRP were detected in the capillary-like structures and in the cytoplasm and nucleus of hfBECs. P-gp protein/*ABCB1* mRNA and BCRP protein levels decreased (*p* ≤ 0.05) in hfBECs, between early and mid-gestation. However, no differences in P-gp or BCRP function or cell proliferation and viability were observed between early and mid-gestation. These novel studies are the first to demonstrate that hfBECs derived in early and mid-gestation express functionally active P-gp and BCRP, which likely contribute to the protection of the fetal brain in very early pregnancy.

The present study localized P-gp and BCRP to microvessels within the human fetal brain (18–20 weeks of gestation). This is consistent with previous observations that showed P-gp and BCRP immunostaining in human fetal brain microvascular endothelial cells [18,39,40,41,42], as well as in fetal brain tissue as early as 6–7 weeks gestation [43]. In addition, previous studies have reported that P-gp and BCRP immunostaining in 22–42-week fetal human brain and in adult brain tissue [44,45].

The localization of vWF, as well as ZO-1 and Cldn-5 proteins, in the isolated hfBECs confirmed the endothelial phenotype of the derived cells. Furthermore, neither early or mid-gestation hfBECs expressed the glial cell marker (GFAP). Many previous studies have demonstrated that under the correct conditions, cultured BECs form capillary-like tubes [46,47,48]. In the present study, the hfBECs derived from early and mid-gestation fetuses formed structures that clearly resembled capillary-like tubes. Importantly, these tube structures expressed the endothelial marker, vWF. Thus, the cells isolated from early and mid-gestation fetal brains clearly characterize as BECs [49,50]. Furthermore, early and mid-gestation hfBECs express the high levels of the drug transporters, P-gp and BCRP.

Endothelial cells produce capillary-like structures in response to angiogenic signals. This process involves the growth, migration and differentiation of endothelial cells, which line the inside wall of blood vessels [51]. Previous studies have reported that the silencing of *ABCB1* (P-gp-encoding gene) reduced invasion and migration and increased the tube formation of extravillous trophoblast cells HTR8/SV_neo_ [52], demonstrating a potential role of P-gp in regulating the tube formation of invading/migrating cells. In addition, P-gp/caveolin-1 interactions can modulate brain endothelial angiogenesis and P-gp-dependent cell migration in RBE4 cells [53]. Together, these studies suggest that P-gp in early hfBECs may be involved in endothelial cell tube formation and the angiogenesis of brain microvessels. Clearly, further studies are required to investigate this possibility.

When analyzing P-gp and BCRP co-localization in mid-gestation hfBECs, we observed that not all cells expressing vWF exhibited high levels of P-gp or BCRP staining. This is in contrast to what was observed in histological sections of brain microvessels, where the majority of cells expressing vWF co-stained with P-gp or BCRP. It has been reported that P-gp and BCRP levels are linked to cell cycle and p53 expression [54,55]. It is possible that in vitro conditions may impose greater differences in cell cycle phases compared to the in vivo conditions and may, therefore, explain the lack of P-gp and BCRP staining in a small number of vWF-positive cells. However, isolated hfBECs exhibited a significant decrease in total P-gp and BCRP protein and *ABCB1* mRNA expression between early and mid-gestation, although there was no change in P-gp or BCRP function. In our previous study, we reported that adult brain endothelial cells have shown a disconnect between changes in function and changes in transporter mRNA and protein [23]. Furthermore, we also reported a significant decrease in *ABCG2* mRNA expression in the mouse fetal brain with advancing gestation (from E15.5 to E18.5) with no alteration in BCRP protein levels [56]. The structure of the human fetal brain is complex and there are significant differences in the structure and function between different species which creates difficulties for cross-species comparisons [57,58]. The discrepancy in the trajectories of BCRP protein and *ABCG2* mRNA expression may be due to the impact of protein stability or delayed protein synthesis, as has previously been reported [59,60,61]. These studies suggest that the regulation of P-gp and BCRP mRNA and protein expression in fetal BBB is gestational age-dependent, though this does not appear to impact baseline P-gp and BCRP efflux function in the human, at least up until mid-pregnancy.

Due to the very limited availability of human fetal brain tissue for research, our sample size was relatively small (*n* = 6/group). However, it is important to point out that a sample size of six per group (or less in specific cases) has been sufficient to show statistical alterations in P-gp protein/*ABCB1* mRNA and BCRP protein/*ABCG2* mRNA levels and/or function in human [16,30,31,53,62,63] and in animal gestational tissues [15,62,63,64]. In the current study, we were unable to collect any clinical information (apart from gestational age) about the elective pregnancy terminations (mandated by our REB). It is possible that differences in clinical conditions or differences in demographics, together with a relatively small sample size, may account for the variability in some of the parameters measured. Notwithstanding, these are novel studies that have not been undertaken previously in this very unique early and mid-gestation sample.

The confocal microscopic analysis of the subcellular compartmentalization of P-gp and BCRP in hfBECs isolated in the second trimester indicated that P-gp and BCRP were distributed across different subcellular fractions. In particular, P-gp and BCRP exhibited strong punctate immunofluorescent staining within the cytosol, particularly in the perinuclear region, possibly indicating localization to the perinuclear organelles, such as endoplasmic reticulum (ER), Golgi apparatus and mitochondria. Unexpectedly, we also noted punctate staining associated with the nucleus. To further define the nuclear localization of P-gp and BCRP, we conducted z-stack microscopy analysis of the early and mid-gestation hfBECs and determined that the punctate staining of P-gp and BCRP was actually within the nucleus and the nuclear membrane. Previous studies have reported P-gp immunostaining and protein expression within the nucleus of LoVo cell and in microvessels within the rat brain [65,66], as well as the cellular/subcellular distribution of P-gp in rat and human brain tissues [67]. Furthermore, BCRP immunostaining has been reported within the nucleus of glioblastoma cells (LN229) and tumor biopsies, in human lung cancer cells (A549) and human alveolar epithelial type 2 cells [68,69,70]. Importantly, we found that BCRP localized to the nuclear compartment of normal (non-malignant) fetal brain endothelial cells. A common feature of hfBECs and cancer cells is the high rate of proliferation. Szaflarski et al. suggested that different P-gp glycoside chains play a role in the intracellular trafficking of P-gp and may determine the distribution of the protein in cells [65]. Furthermore, it has been suggested that the PI3K-Akt signaling pathway regulates the relative expression of BCRP in the cell surface in the LLC-PK1 kidney cell line [71]. In lung cancer cells, BCRP is enriched inside the nucleus and binds to the E-box region of the E-cadherin (CDH1) gene promoter to regulate its transcription [70]. We also detected P-gp and BCRP in the nuclear membrane of early and mid-gestation hfBECs. It is possible that nuclear P-gp and BCRP may provide an efflux function removing substances from the nucleus, thus protecting nuclear DNA from potential damaging effects. This is consistent with the fact that BCRP knockdown in drug resistant cancer cells increased levels of intracellular reactive oxygen species and apoptotic rates in these cells [72].

The presence of P-gp and BCRP in organelles is consistent with the fact that P-gp is distributed in caveolae, cytoplasmic vesicles, Golgi complex, and rough endoplasmic reticulum (RER) in rat and human brain tissues [67]. Furthermore, Takada and colleagues found BCRP to be present within mitochondria and in the perinuclear region of various cell lines, including MDCKII, MDCK-BCRP, IGROV1 and T8 cells [73]. Further studies are required to define which organelles contain P-gp and BCRP within hfBECs.

We further analyzed the relative levels of P-gp and BCRP level in subcellular compartments of hfBECs isolated in early and mid-gestation. Interestingly, there was a significant decrease in P-gp levels in the plasma membrane and nuclear fractions from early to mid-gestation, with no changes within cytosol and organelles subcellular fractions. Although we did find a significant decrease in total BCRP protein in the hfBECs between early and mid-gestation, we did not identify a significant reduction in BCRP in any of the subcellular fractions. However, there were tendencies for reductions in BCRP in both the cytosolic and nuclear fractions. Subcellular extractions require substantial additional processing and it is possible that there may have been a variable loss of protein in some of the samples leading to increased variation within the groups. In this connection, the variability in BCRP protein in the total protein fraction is lower than in the subcellular fractions. Nevertheless, these studies confirmed the expected presence of P-gp and BCRP within the plasma membrane fractions, but also their presence within the cytosol, organelles and the nucleus. Importantly, the organelle and nuclear fractions were also positive for Na^+^/K^+^-ATPase, raising the possibility that P-gp and BCRP may be present within the membrane fractions of these structures. These studies suggest that the regulation of P-gp or BCRP subcellular distribution in fetal BECs is gestational age-dependent. Since the investigation of the role of nuclear P-gp and BCRP in cellular physiology is a novel and emerging research area, it is not possible to be definitive about the biological significance of the gestational age dependent variations in nuclear P-gp herein observed. However, it is tempting to speculate that the increasing rates of CNS maturation, consisting of the specific developmental windows of intense neurogenesis, neuronal migration, synaptogenesis and myelination from early gestation on [74] have some influence on P-gp levels in the nucleus of hfBECs. Further studies are required to determine the functional consequences of such changes.

## 5. Conclusions

We have characterized endothelial cells isolated from human fetal brains from early and mid-gestation. These cells express functionally competent drug transport proteins P-gp and BCRP, which are known to contribute to the BBB, and thus protect the fetal brain from potential toxins and drugs that might impact the development of the fetal CNS. We found a reduction in the expression of these drug transporters between early and mid-gestation. We also identified the presence of these proteins in the plasma membrane, and that P-gp plasma membrane (but not BCRP) levels decrease in second trimester, but without affecting P-gp efflux function. We also described their presence in subcellular fractions, including the nucleus and that P-gp (but not BCRP) expression in the nucleus was reduced in the second trimester. The significance of the nuclear expression of the proteins in the nucleus remains to be determined but could potentially impact exposure to chemicals that could potentially damage DNA within these cells. The hfBECs characterized in this study will allow detailed investigation of the role and regulation of drug transporters and other factors in the development and protection of the human fetal brain across the first half of pregnancy.

## Figures and Tables

**Table 1:** List of primers used in this study.

Primer Name	Sequence	Reference
*ABCB1*	Forward: 5′GCCCTTGTTAGACAGCCTCA-3′	[30]
	Reverse: 5′GGCTTTGTCCAGGGCTTCTT-3′	
*ABCG2*	Forward: 5′-TGGAATCCAGAACAGAGCTGGGGT-3′	[30]
	Reverse: 5′-AGAGTTCCACGGCTGAAACACTGC-3′	
*YWHAZ*	Forward: 5′-CCGCCAGGACAAACCAGTAT-3	[33]
	Reverse: 5′-CAC ATC ACA GCT CCC CAC CA-3′	
*TOP1*	Forward: 5′-GATGAACCTGAAGATGATGGC-3′	[33]
	Reverse: 5′-TCAGCATCATCCTCATCTCG-3′	

## Data Availability

The data that support the findings of this study are available from the corresponding author upon reasonable request.

## Supplementary Materials

The following supporting information can be downloaded at: https://www.mdpi.com/article/10.3390/cells11142259/s1, Figure S1: Expression of von Willebrand Factor (vWF) in human fetal brain microvessels. Figure S2: Expression of Zonula Occludens-1 (ZO-1) and Claudin-5 (Cldn-5) tight-junction proteins in early and mid-gestation primary human fetal brain endothelial cells (hfBECs). Figure S3: Inhibition of P-glycoprotein (P-gp) and Breast Cancer Resistance Protein (BCRP) increases substrate accumulation in early- and mid-gestation primary human fetal brain endothelial cells (hfBECs). Figure S4: Inhibition of P-glycoprotein (P-gp) function increases Rhodamine 123 (Rho123) accumulation in early- and mid-gestation primary human fetal brain endothelial cells (hfBECs). Figure S5: Human fetal brain endothelial cells (hfBECs) proliferation and viability does not change from early- to mid-pregnancy.
