# Supplementary material for: Functional Expression of Multidrug-Resistance (MDR) Transporters in Developing Human Fetal Brain Endothelial Cells
Source: Cells. 2022 Jul 21;11(14):2259. doi: 10.3390/cells11142259 (PMC9323234; doi:10.3390/cells11142259)
Supplement: Supplementary file 1 [file cells-11-02259-s001.zip › cells-1795618-supplementary.pdf]

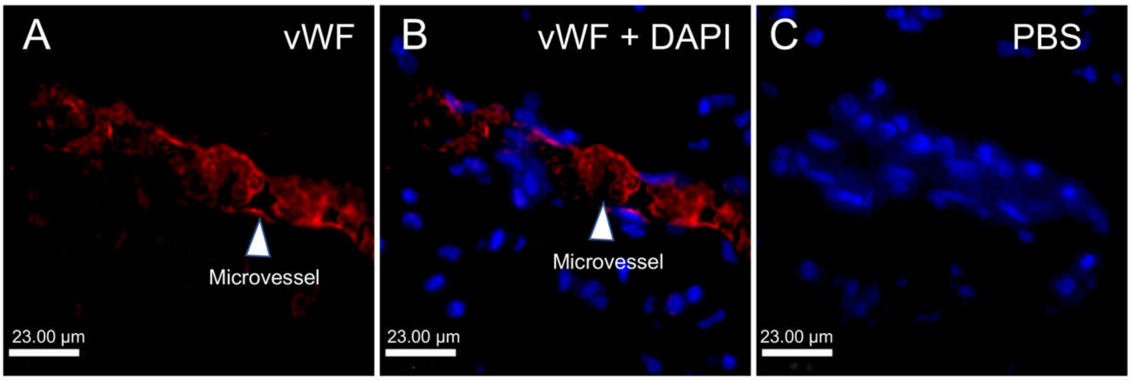

Supplementary Figure S1: Expression of von Willebrand Factor (vWF) in human fetal brain microvessels. Representative immunofluorescence **(A-C)** expression of the endothelial cell marker vWF (red) identified the human fetal brain microvessels at 18-20 weeks of pregnancy. **(B-C)** DAPI (blue; a nuclear marker). **(A)** vWF only, **(B)** vWF and DAPI. **(C)** PBS (negative control). N=4. Scale bar = 23 µm.

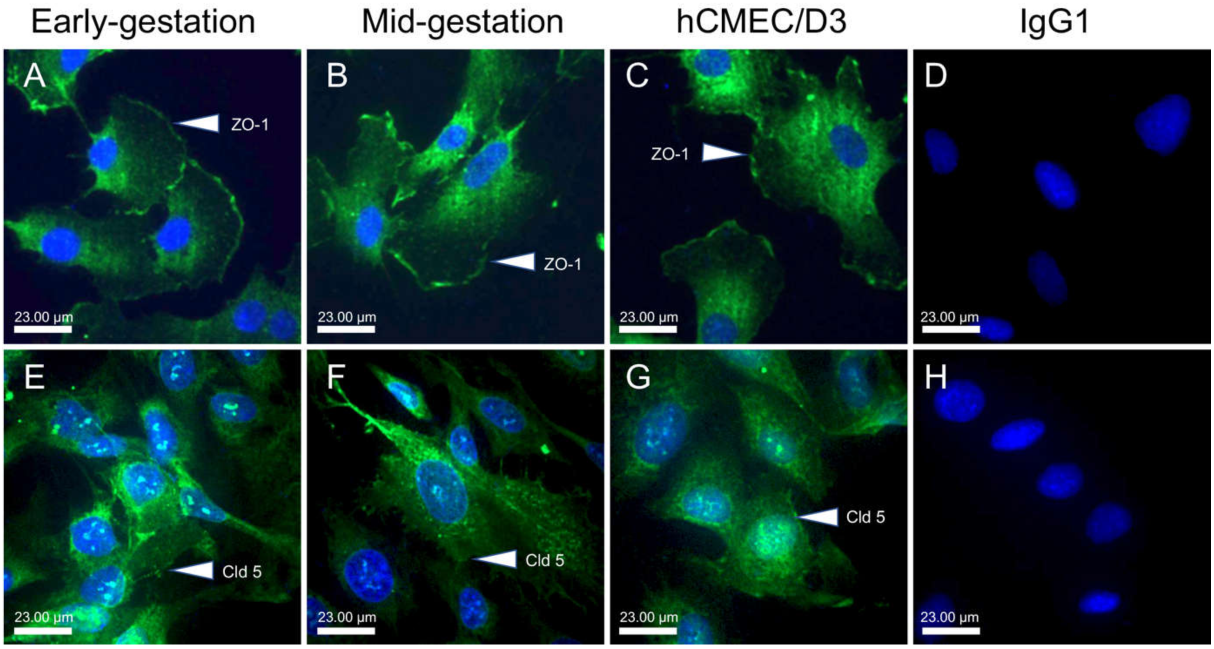

Supplementary Figure S2: Expression of Zonula Occludens-1 (ZO-1) and Claudin-5 (Cldn-5) tight-junction proteins in early- and mid-gestation primary human fetal brain endothelial cells (hfBECs). (A-C) Representative immunofluorescence of ZO-1 (green) and **(E-G)** Cldn-5 (green) in cytoplasm and plasma membrane of adjoining endothelial cells **(A,E)** first trimester hfBECs, **(B,F)** second trimester hfBECs, **(C,G)** adult endothelial cell line (hCMEC/D3), and (**D,H)** IgG1 isotype control. **(A-H)** DAPI (blue; a nuclear marker). N=4. Scale bar = 23 µm.

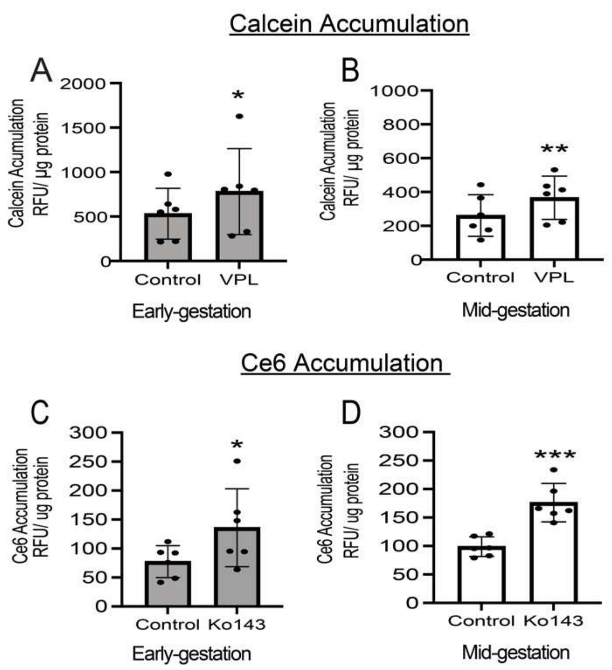

Supplementary Figure S3. Inhibition of P-glycoprotein (P-gp) and Breast Cancer Resistance Protein (BCRP) increases substrate accumulation in early- and mid-gestation primary human fetal brain endothelial cells (hfBECs). (A-B) The presence of the P-gp inhibitor verapamil (VPL) increased Calcein-AM accumulation (a P-gp substrate) in all cell systems (early and mid-getsation), indicating reduced P-gp function and validating the Calcein-AM assay. Control cells were exposed to 10^{-4} methanol (VPL vehicle). **(C-D)** The presence of the BCRP inhibitor Ko143 increased Ce6 accumulation (a BCRP substrate) in all cell systems, indicating reduced BCRP function and validating the Ce6 assay. Control cells were exposed to 10^{-5} M ethanol (Ko143 vehicle). Data are expressed as mean ± SD. N=6/gp. Statistical differences were conducted using a paired *t*-test. **p*<0.05, ** *p*<0.01, ****p*<0.001.

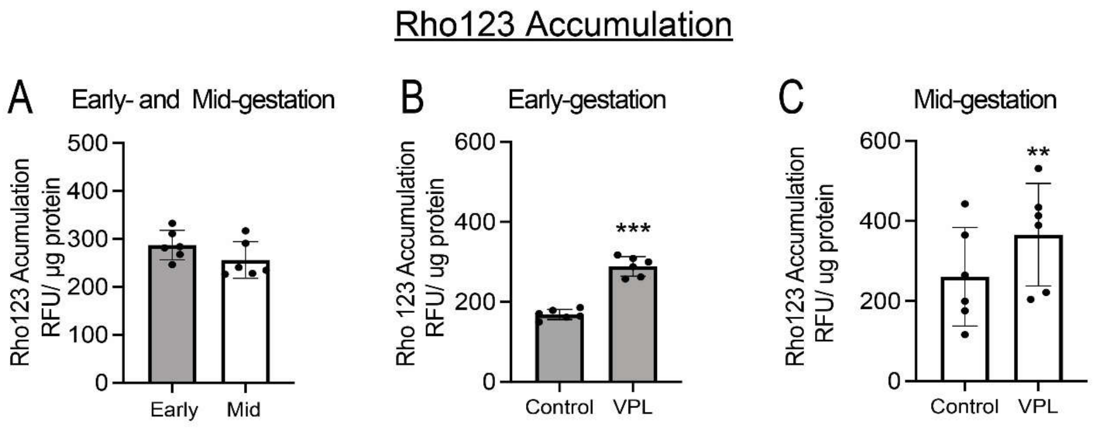

Supplementary Figure S4. Inhibition of P-glycoprotein (P-gp) function increases Rhodamine 123 (Rho123) accumulation **in early- and mid-gestation primary human fetal brain endothelial cells (hfBECs). (A)** Rho123 (an alternative P-gp substrate) accumulation in early and mid-gestation hfBECs. The presence of the P-gp inhibitor VPL increased Rho123 in **(B)** early gestation and **(C)** mid-gestation hfBECs, indicating reduced P-gp function and validating the P-gp functional assay. Control cells were exposed to 10^{-4} M ethanol (VPL vehicle). Data are displayed as mean ± SD. N=6. Statistical differences were tested using (A) unpaired t-test (B,C) paired *t*-test. ***p*<0.01, ****p*<0.001

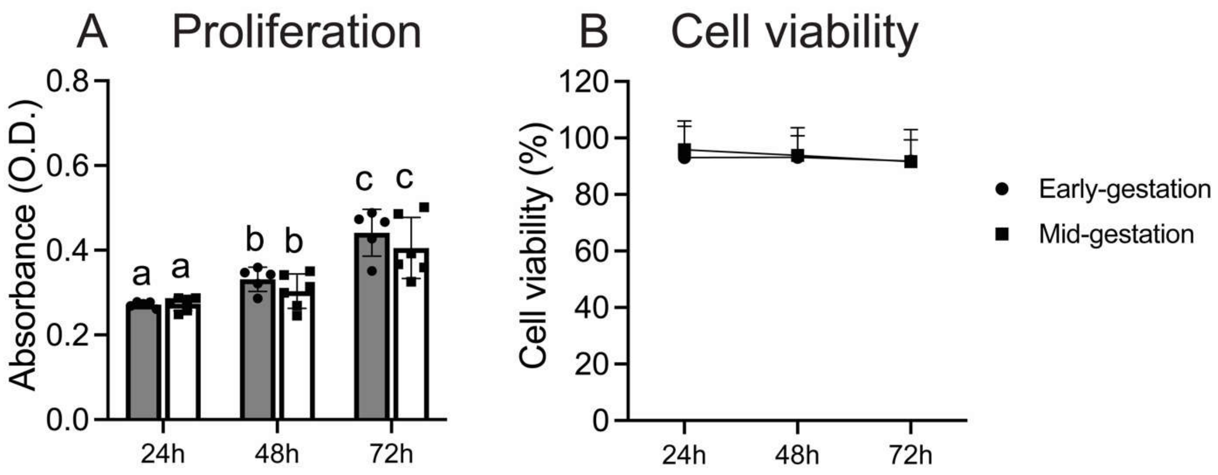

Supplementary Figure S5. Human fetal brain endothelial cells (hfBECs) proliferation and viability does not change from early- to mid-pregnancy. (A) Cell proliferation of early (N=5) and mid- (N=6) pregnancy hfBECs during three consecutive days. There was no group effect, but an effect of time (24-72h). **(B)** Cell viability of early (N=5) and mid- (N=6) pregnancy hfBECs during 3 consecutive days. There were no group or time differences during three consecutive days. Data are displayed as mean ± SD. Statistical differences were tested using Two-Way Repeated-Measures ANOVA followed by Sidak's multiple comparisons test. Different letters indicate significant differences ($P<0.01$) in proliferation between days in culture.
